# Oxidative Stress Response Is Mediated by Overexpression and Spatiotemporal Regulation of Caveolin-1

**DOI:** 10.3390/antiox9080766

**Published:** 2020-08-18

**Authors:** Andreas Goutas, Ioanna Papathanasiou, Evanthia Mourmoura, Konstantinos Tsesmelis, Aspasia Tsezou, Varvara Trachana

**Affiliations:** 1 Laboratory of Biology, Faculty of Medicine, School of Health Sciences, University of Thessaly, Biopolis, 41500 Larissa, Greece; agoutas@uth.gr (A.G.); konstantinos.tsesmelis@uni-ulm.de (K.T.); atsezou@med.uth.gr (A.T.); 2 Laboratory of Cytogenetics and Molecular Genetics, Faculty of Medicine, School of Health Sciences, University of Thessaly, Biopolis, 41500 Larissa, Greece; iopapat@med.uth.gr (I.P.); emourmoura@med.uth.gr (E.M.)

**Keywords:** caveolin-1, genotoxicity, oxidative stress, senescence, osteoarthritis

## Abstract

Oxidative stress (OS) has been linked to the aetiology of many diseases including osteoarthritis (OA). Recent studies have shown that caveolin-1—a structural protein of plasma membrane’s caveolae—is upregulated in response to OS. Here, we explore the function of caveolin-1 in chondrocytes derived from healthy individuals (control) and OA patients that were subjected to exogenous OS. We showed that caveolin-1 was upregulated in response to acute OS in the control, but not in OA chondrocytes. Moreover, OS-induced DNA damage analysis revealed that control cells started repairing the DNA lesions 6 h post-oxidative treatment, while OA cells seemed unable to restore these damages. Importantly, in the control cells, we observed a translocation of caveolin-1 from the membrane/cytoplasm in and out of the nucleus, which coincided with the appearance and restoration of DNA lesions. When caveolin-1 was prevented from translocating to the nucleus, the control cells were unable to repair DNA damage. In OA cells, no such translocation of caveolin-1 was observed, which could account for their inability to repair DNA damage. Taken together, these results provide novel insights considering the role of caveolin-1 in response to OS-induced DNA damage while revealing its implication in the pathophysiology of OA.

## 1. Introduction

The imbalance between the production and scavenging of reactive oxygen species (ROS) is referred to as oxidative stress (OS). OS jeopardizes cellular homeostasis mainly by putting at risk the integrity of macromolecules [1]. Damage to the macromolecules, especially DNA, is considered as a causal factor for cellular senescence and organismal ageing and has been associated with several age-relates diseases [2]. Osteoarthritis (OA), the most common age-related disease [3], has been linked to chondrocyte senescence as well as to OS [4,5]. Furthermore, as our group and others have demonstrated, OS could also lead to mitochondrial dysfunction, which will in turn amplify ROS accumulation, comprising a vicious loop that promotes OA pathogenesis [6,7].

Previous studies examining OS response have revealed that sublethal doses of exogenous oxidative insult results in upregulation of caveolin-1 protein levels [8,9]. Caveolin-1 is the major component of caveolae, the omega-shaped plasma membrane invaginations [10]. Its caveolin scaffolding domain (CSD) gives it the ability to act as a scaffold protein and interact with various molecules, therefore mediating several processes such as caveolae homeostasis, signal transmission, and oncogenesis [11]. Caveolin-1 has also been associated with cellular senescence [12,13], while its expression has been reported to be significantly increased in the brain, spleen, and lung of old rats [12]. However, other observations contradict the above results: knockdown of caveolin-1 in human endothelial cells resulted in senescence-related morphological changes [14], and in mouse embryonic fibroblasts led to mitochondrial dysfunction, which is known to be linked to stress-induced senescence [15].

Similar paradoxical observations considering caveolin-1 have been reported in OA. Dai and colleagues demonstrated that catabolic stress induces senescence in chondrocytes via upregulation of caveolin-1 [16], whereas others have reported significantly decreased expression of caveolin-1 in the cartilage tissues of individuals suffering from severe OA [17]. Additionally, caveolin-1 upregulation has been linked to cancer therapy resistance [18], which is suggested to result from its implication in DNA damage repair [19]. It was demonstrated that when caveolin-1 expression is silenced, the levels of γ-H_2_AX were significantly higher following IR-irradiation [19]. The γ-phosphorylation of H_2_AX (at Ser13) is well documented to take place upon the formation of double strand breaks (DSB), regardless of the different means that induce DSB. DSB activate ATM/ATR that bind to the DNA and induce phosphorylation of many downstream proteins such as H2A.X and 53BP1, in order to both mark the location and initiate repair of the damage [20]. DSB can be generated by a wide variety of factors that include ionizing radiation and radiomimetic agents, but also OS [21]. While it is known that the most common oxidative DNA lesions are abasic sites and single strand DNA breaks (SSB), DSB can also be formed as a result of elevated ROS. Specifically, it has been demonstrated that exposure to H_2_O_2_ results in the formation of 8-oxo-2′-deoxyguanosine (8-oxo-dG), a major product of DNA oxidation [22]. 8-oxo-dG lesions could lead to reduced efficiency of the base excision repair process leading, in turn, to persistent DNA single strand break (SSB) intermediates; the latter could then act as double strand break (DSB)-prone sites in subsequent rounds of DNA replication [23]. Our group has, as a matter of fact, previously demonstrated that exogenous induction of OS via H_2_O_2_ treatment leads to γ-H_2_AX labelled DSB in primary embryonic lung fibroblasts [24], mesenchymal stem cells [25] as well as in chondrocytes [7]. A γH2AX focus, representing a DSB, will appear within minutes from the lesion formation and then decline and eventually disappear after approximately 24 h, following the rate of DSB repair [26]. By monitoring these kinetics after H_2_O_2_ treatment, we also demonstrated an attenuated ability of senescent/OA cells to repair these DNA lesions [7,24,25].

Given all the above, we aimed at exploring the function of caveolin-1 in response to oxidative DNA damage. We first aimed at eliminating the discrepancies regarding caveolin-1 expression levels by analysing its levels under normal conditions as well as under exogenously induced OS, both in normal and OA chondrocytes. Furthermore, we explored caveolin-1 possible implications in regulating the formation and restoration of H_2_O_2_-induced γH_2_AΧ-labeled DNA damage lesions. We found that caveolin-1 mRNA and protein levels were significantly elevated in OA chondrocytes compared to the control cells under normal conditions. Our results indicate that this increase could account for the failure of OA cells to further upregulate caveolin-1 levels in response to acute exogenous oxidative insult, as the control cells did, and possibly with their inability to repair OS-derived DSB. We further report, for the first time to our knowledge, an OS-induced caveolin-1 translocation in and out of the nucleus, in the control, but not in OA chondrocytes, which coincides with the appearance and restoration of DSB. We therefore suggest that the observed here OA chondrocytes’ inability to increase caveolin-1 levels and spatiotemporally regulate it in response to OS contributes to the disease aetiopathogenesis.

## 2. Materials and Methods

### 2.1. Cartilage Samples

Articular cartilage samples were obtained from femoral condyles of patients with primary osteoarthritis, who had undergone knee replacement surgery at the Orthopaedics Department of the University Hospital of Larissa, Larissa, Greece. A total of 17 patients were included in this study (13 females/four males; mean age 68.13 ± 6.37 years). Before surgery OA patients were graded using radiography according to the Kellgren–Lawrence (K/L) system [27] and all of them had a K/L score >2. The assessment of the radiographs by two independent expert observers was blinded. We also obtained healthy articular cartilage from 13 individuals, eight females, and five males, mean age 57.91 ± 5.15 years, who had undergone knee fracture repair surgery or amputation surgery with no previous history of OA. Patients with rheumatoid arthritis or other autoimmune disease as well as chondrodysplasias, infection induced OA, or post-traumatic OA were excluded from the study. All individuals involved in this study gave informed consent. The protocol was approved by the local ethical committee of the University Hospital of Larissa, which follows the ethical guidelines of the 1975 Declaration of Helsinki. The code from the Ethics Committee of the University Hospital of Larissa is: Νο 55277, 15/11/2016.

### 2.2. Primary Cultures of Articular Chondrocytes and Oxidative Stress (OS) Treatment

Articular cartilage from OA patients (hereafter OA) and healthy individuals (hereafter control) was subjected to sequential digestion with 1 mg/mL pronase for 30 min and 1 mg/mL collagenase P (Roche Applied Science, Mannheim, Germany) overnight at 37 °C. Cell cultures of isolated chondrocytes from individual specimens was performed as described previously [7] in Dulbecco’s Modified Eagles Medium/Ham’s F-12 (DMEM/F-12) (GIBCO, Life Technologies, Paisley, UK), supplemented with 10% fetal bovine serum (FBS, Sigma-Aldrich, St Louis, MI, USA), penicillin (100 IU/mL), and streptomycin (100 μg/mL) in a humidified incubator set to 37 °C, 5% CO_2_ until reaching confluence in 4–6 days. To avoid dedifferentiation of chondrocytes, for all the analyses, we used cultured primary chondrocytes at passage 1 only.

Exposure of OA and the control chondrocytes to exogenous acute oxidative stress (OS) was performed as described in our previous studies [7,25] with some modifications. Specifically, 100,000 chondrocytes from six OA patients and six healthy individuals were seeded in each well of a 6-well plate and at around 70% confluency, they were exposed to 200 μM H_2_O_2_ for 30 min in a serum free-medium. For filipin pre-treatment, cells from three healthy individuals were incubated for 24 h in complete medium containing 1 μg/mL of filipin and then subjected to the oxidative insult described above. After the OS treatment, cells were left to recover in fresh complete medium for different lengths of time (1 h, 3 h, and 24 h of recovery time for western blot analyses and 1 h, 3 h, 6 h, 24 h, and 48 h of recovery time for fluorescent microscopy analysis, see below “Immunofluorescence”). For DNA damage generation with a different agent, bleomycin, a radiomimetic chemotherapeutic drug, (kind gift from Dr. K. Dimas, Dept. Pharmacology, Faculty of Medicine, University of Thessaly, Larissa, Greece) was used at 0.2 U/mL for two different lengths of time (6 h and 12 h).

### 2.3. Total Reactive Oxygen Species (ROS)/Superoxide Detection

Chondrocytes (100,000) from three OA patients and three healthy individuals were seeded in each well of a 6-well plate for 24 h. The ROS Inducer Pyocyanin (PYO) [28] was added to a final concentration of 200 μM for 30 min to one of the control samples in order to serve as a positive control. Oxidative stress was detected by staining with two fluorescent dyes from the ROS-ID^®^ Total ROS/Superoxide Detection Kit (ENZ-51010, Enzo, Farmingdale, NY, USA). The intensity of the green dye (total ROS detection reagent) represents the level of oxidative stress and the intensity of the orange dye (superoxide detection reagent) provides exclusive detection of the superoxide.

### 2.4. RNA Extraction and Quantitative Real-Time PCR (qRT-PCR)

Trizol reagent (Invitrogen, Life Technologies, Paisley, UK) was used for total RNA isolation according to the manufacturer’s instructions. Preservation of 28S and 18S ribosomal RNA (rRNA) species was used to assess the RNA integrity and all samples used in the study were prominent with 28S and18S rRNA components. The yield was quantified spectrophotometrically. Transcription of 1 μg RNA to complementary cDNA (cDNA) was performed using SuperScript III reverse transcriptase (Invitrogen, Life Technologies, Paisley, UK) and random primers (Invitrogen, Life Technologies, Paisley, UK). Quantification of caveolin-1 mRNA expression was performed by real-time time Polymerase Chain Reaction (qRT-PCR, ABI 7300; Applied Biosystems, Foster City, CA, USA). Primers used for caveolin-1 (accession number NM_001753) were: 5′-TAATCCAAGCATCCCTTTGC-3′ (forward) and 5′-AAAGTCCCCAAAGGCAGAAT-3′ (reverse). For reference gene GAPDH primers were: 5′-GAGTCAACGGATTTGGTCGT-3′ (forward) and 5′-GACAAGCTTCCCGTTCTCAG-3′ (reverse). Each analysis was performed in triplicates using 2 μL of cDNA per reaction for each sample (17 OA samples and 13 control samples). Calculations were performed based on the Ct method, which was previously described [29]. The formula 2^[−ΔΔCt] is used to calculate the expression of target genes normalized to a calibrator (a control sample). The Ct data for target gene and housekeeping gene were used to establish ΔCt values [ΔCt = Ct (target gene) − Ct (housekeeping gene)] in each sample (control or OA). Ct values in our experiments were normalised against the endogenous reference (GAPDH) (ΔCt = Ct target − Ct GAPDH) and ΔΔCt values were calculated by subtracting the calibrator from the ΔCt value of each target. The relative quantity (RQ), or Nº fold, was calculated using the equation RQ = 2 −ΔΔCt.

### 2.5. Protein Extraction and Western Blot Analyses

Chondrocytes from 17 OA patients and 13 healthy individuals were lysed using Radioimmunoprecipitation assay (RIPA) buffer with the addition of 10 mM Tris (pH 7.5), 150 mM NaCl, 1% Triton X-100, 1% sodium deoxycholated, 0.1% sodium dodecyl sulfate (SDS), 1 mM Ethylenediaminetetraacetic acid (EDTA) and supplemented with protease inhibitor cocktail (Roche Applied Science, Mannheim, Germany). Protein concentrations were quantified using bovine serum albumin (BSA) as the standard and the Bio-Rad Bradford protein assay protocol (Bio-Rad Protein Assay, BioRad, Hercules, CA, USA). A total of 20 μg of total protein were analysed in 12% sodium dodecyl sulfate–polyacrylamide gel electrophoresis (SDS-PAGE) gel and transferred to Polyvinylidene difluoride (PVDF) (Millipore, Billerica, MA, USA) using the Transblot SD semi-dry Transfer Cell (BioRad, Hercules, CA, USA). A total of 5% *w/v* non-fat dry milk in PBS/0.1%Tween^20^ was used to block the membranes. Subsequently, the membranes were probed with antibodies against caveolin (1:1000 dilution, #A1915, rabbit polyclonal, Santa Cruz Biotechnology Inc., Dallas, TX, USA) and p16 (1:1000 dilution, p16 INK4A (D3W8G), rabbit monoclonal, Cell Signalling Technology, Danvers, MA, USA) overnight at 4 °C. Equal protein loading was verified using anti-Glyceraldehyde 3-phosphate dehydrogenase (GAPDH) rabbit polyclonal antibody (1:3000, G9545, Sigma-Aldrich, St. Louis, MO, USA). Membranes were then probed with the appropriate secondary antibody (anti-rabbit) conjugated with horseradish peroxidase (1:10,000, Invitrogen, Life Technologies, Paisley, UK) for 1 h at room temperature. Enhanced chemiluminescence was detected using Enhanced Chemiluminescence (ECL) substrates (Thermo Fisher Scientific Inc., Waltham, MA, USA)). Each western blot analysis was performed at least three times. Western blot images were taken using an Uvitec Cambridge Chemiluminescence Imaging System. ImageJ (1.47r, Wayne Rasband National Institutes of Health, Bethesda, MD, USA [30] was used to analyse the expression of caveolin-1/p16, normalised relatively to the housekeeping protein (GAPDH) at each time point, based on band density.

For quantification of the levels of caveolin-1 following the OS treatment described above (in “Primary cultures of articular chondrocytes and OS treatment”), 100,000 chondrocytes from six different OA patients and six different healthy individuals were lysed in RIPA buffer and the same western blot procedure was followed.

### 2.6. Immunofluorescence

Immunofluorescence experiments were performed using chondrocytes from three OA patients and three healthy individuals and repeated three times, as previously described [24] with slight modifications. In particular, 100,000 chondrocytes grown on coverslips in 6-well plates, subjected to the above described exogenous OS and left to recover for different lengths of time (1 h, 3 h, 6 h, 24 h, and 48 h of recovery time) were fixed in 4% paraformaldehyde for 20 min at Room Temperature (RT). After blocking in phosphate buffer saline (PBS) containing 0.02% Tween20 and 1% BSA for 10 min, coverslips were incubated with anti-caveolin-1 (1:500, #A1915, rabbit polyclonal, Santa Cruz Biotechnology Inc., Dallas, TX, USA) for 1 h at room temperature (RT). Subsequently, the coverslips were incubated for 1 h with the appropriate fluorescent dye-conjugated secondary antibody (1:500 dilution, Alexa Fluor 594, Molecular Probes). For DSB detection, fixed slides were incubated with mouse monoclonal anti-γH2AX (1:200 dilution, 05-636, clone JBW301, Millipore, Burlington, MA, USA), 53BP1 (1:500, clone BP13, Millipore, Burlington, MA, USA) and the corresponding secondary antibodies. Oxidative DNA damage was detected using the 8-oxo-dG antibody (1:200, Clone 2E2, Trevigen, Gaithersburg, MD, USA) and appropriate secondary antibody. Vectashield mounting medium (Vector Laboratories, Burlingame, CA, USA) containing 4,6-diamidino-2-phenylindole (DAPI) was used to visualise the nuclei/micronuclei. A ZEISS Axio Imager Z2 fluorescent microscope and A ZEISS LSM 800 confocal microscope were used for image capture. Images were then analysed with ImageJ software (1.47r, Wayne Rasband National Institutes of Health, Bethesda, MD, USA [30]. At least five randomly selected fields were analysed by two independent observers blinded to the origin of the sample (control or OA chondrocytes) for each time point. Each observer counted at least 200 cells for each time point for each sample used (three control samples and three OA samples) and the means of their counts were used for the statistical analysis.

### 2.7. Statistical Analysis

SPSS 24 software (IBM Corp., Armonk, NY, USA) was used to analyse the data. Statistical significance was determined using the Mann–Whitney U test or Student *t*-test where appropriate. For all comparisons, *p* values less than 0.05 were considered statistically significant and the significance on the graphs is marked with asterisks (* *p* < 0.05; ** *p* < 0.01; *** *p* < 0.001, **** *p* < 0.0001) when a comparison was made between the control and OA cells, or with hashes (# *p* < 0.05; ## *p* < 0.01; ### *p* < 0.001, #### *p* < 0.0001) when comparisons were made between each time point vs. the No Treatment (NT) condition. Results are reported as the mean ± standard error (means ± S.Ε). For results that are presented using box plots, the horizontal lines inside the boxes denote the medians and the horizontal borders of the boxes the interval between the 25th and 75th percentiles. The whiskers show the minimal and maximal values.

## 3. Results

### 3.1. Caveolin-1 Protein and mRNA Levels in Control and OA Chondrocytes

**Figure 1:**
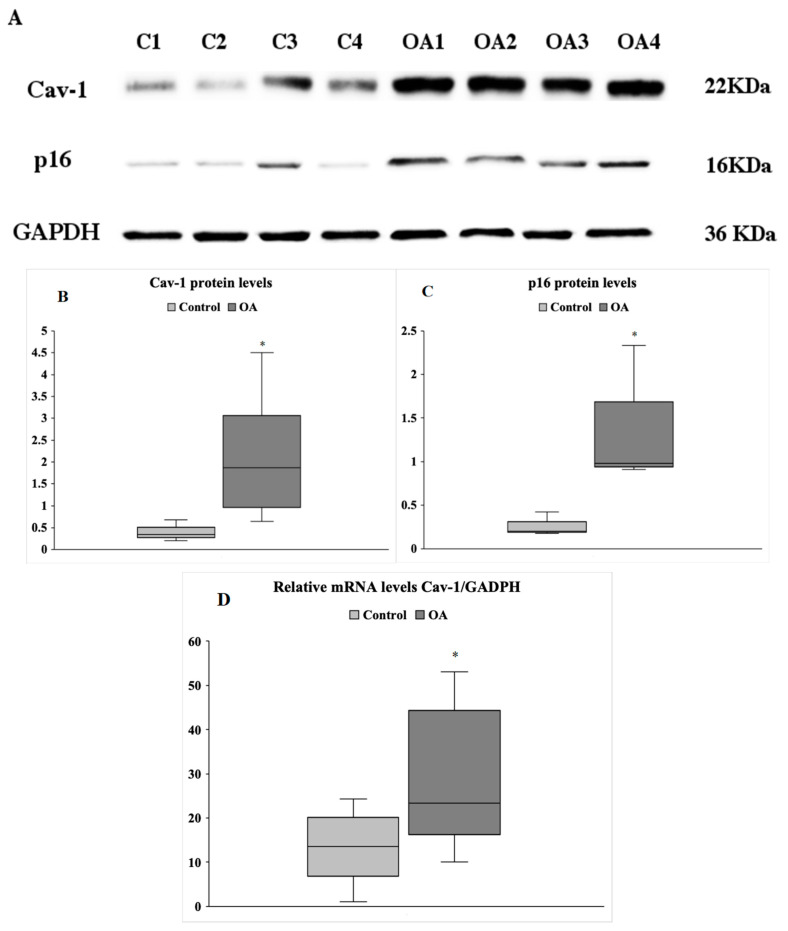
(**A**) Caveolin-1 levels are significantly higher in osteoarthritic (OA) than in control chondrocytes under normal conditions. Characteristic blot showing caveolin-1(Cav-1) and p16 levels in four control and four OA chondrocytes lysates. GAPDH was used as loading control. (**B**) Box blot demonstrating quantification of relative protein expression levels of caveolin-1 in cell lysates from 13 control and 17 OA patients samples was performed based on band density using Image J. (**C**) Box blot demonstrating quantification of relative protein expression levels of p16 in cell lysates from 13 control and 17 OA patients samples was performed based on band density using ImageJ. Values shown are the means ± S.E. * *p* < 0.05 vs. control. (**D**) Box blot demonstrating quantification of relative mRNA levels of caveolin-1 in control and OA chondrocytes. The horizontal lines inside the boxes denote the medians and the horizontal borders of the boxes the interval between 25th and 75th percentiles. The whiskers show the minimal and maximal values. Analysis was performed using chondrocytes from 13 healthy donors and 17 OA patients. * *p* < 0.05 vs. control. *p* values were calculated using the Mann–Whitney U test.

The transcriptional levels of caveolin-1 were evaluated by qRT-PCR in chondrocytes of healthy individuals (control) and OA patients (OA) (13 controls and 17 OA). Caveolin-1 protein expression in the same samples was also analysed by western bot. Figure 1A represents a characteristic image showing the differences in caveolin-1 levels between four different control and four different OA samples (Figure 1A). Calculations of the protein amount based on band density of the total samples analysed (13 control and 17 OA) revealed that caveolin-1 protein levels were increased in OA patient chondrocytes compared to the control cells under normal conditions (Figure 1B, left panel). Similarly, caveolin-1 mRNA levels were significantly upregulated in OA compared to the control chondrocytes (Figure 1D). Additionally, the p16 levels were higher in OA samples than in the control cells (Figure 1A,B, right panel). Furthermore, the increase in p16 levels appeared to positively correlate with the upregulated levels of caveolin-1 in the same samples (Figure 1A), which further indicated the senescent status of OA chondrocytes.

### 3.2. Caveolin-1 Protein Levels in Control and Osteoarthritis (OA) Chondrocytes after Genotoxic Stress

**Figure 2:**
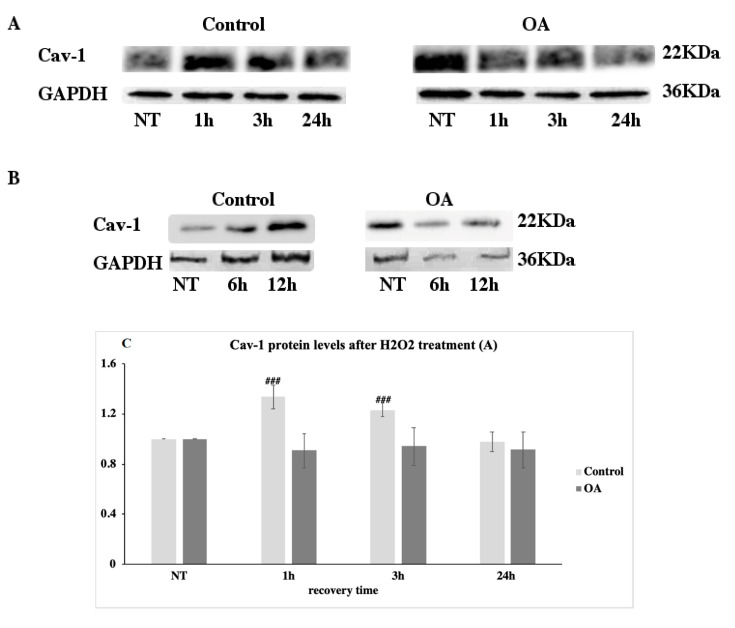

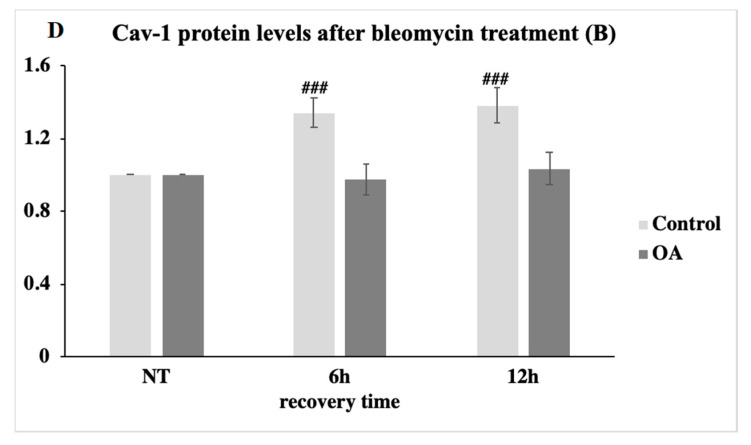
No alterations in caveolin-1 protein levels in osteoarthritic chondrocytes after exogenous oxidative insult. (**A**) Cell lysates of six control and six OA chondrocytes that have been exposed to H_2_O_2_ and left to recover for 24 h were analysed at four different time points (NT, 1 h, 3 h, and 24 h/NT = No Treatment, 1 h = 1 hour, 6 h = 6 hour, and 24 h = 24 hour of recovery time post-treatment) by western blotting using the anti-caveolin-1 (Cav-1) antibody. Antibody against GAPDH was used as loading control. (**B**) Cell lysates of the control and OA chondrocytes that have been exposed to bleomycin for 6 h and 12 h were analysed with western blotting using anti-caveolin-1 (Cav-1) antibody. GAPDH was used as the loading control. (**C**) Quantification of relative protein expression levels of caveolin-1 in cell lysates from control (control) and osteoarthritic chondrocytes (OA) after being exposed to H_2_O_2_ was performed based on band density using ImageJ. (**D**) Quantification of the relative protein expression levels of caveolin-1 in cell lysates from the control and OA chondrocytes after being exposed to bleomycin was performed based on band density using ImageJ. To calculate the differences of protein expression between each time point vs. the No Treatment (NT) condition, protein expression levels at NT were arbitrary set to 1. Analyses were performed three times using cell lysates from six healthy donors and six OA patients. Values shown are the means ± S.E. ### *p* < 0.001 vs. the NT condition. *p* values were calculated using the Student *t*-test.

Caveolin-1 protein levels were also analysed in six control and six OA chondrocytes after being exposed to exogenous oxidative stress and left to recover for different lengths of time, as described in the Materials and Methods. It was observed that in the control cells, caveolin-1 protein levels were upregulated to statistically significant higher values at 1 and 3 h post-treatment compared to the non-treated (NT) condition and decreased back to basal levels after 24 h in recovery. In contrast, in OA chondrocytes, the levels of caveolin-1 did not show any differences between the NT condition and any time point post-treatment (Figure 2A,C).

In order to evaluate whether the upregulation in caveolin-1 levels is linked to DSB formation, we used bleomycin, a radiomimetic drug that also causes DSB [31]. Treatment of the same control and OA samples with bleomycin also increased caveolin-1 protein levels in the control cells, but no significant changes were observed in OA chondrocytes (Figure 2B,D).

These results suggest that caveolin-1 overexpression is part of the chondrocytes’ response to stress-induced DNA damage, which does not seem to occur in cells derived from OA patients.

### 3.3. Microscopic Detection of γH_2_AΧ and Caveolin-1 Localization in Control and OA Chondrocytes after H_2_O_2_ Treatment

#### 3.3.1. Assessment of DNA Double Strand Breaks (DSB) in Control and OA Chondrocytes after OS Treatment

**Figure 3:**
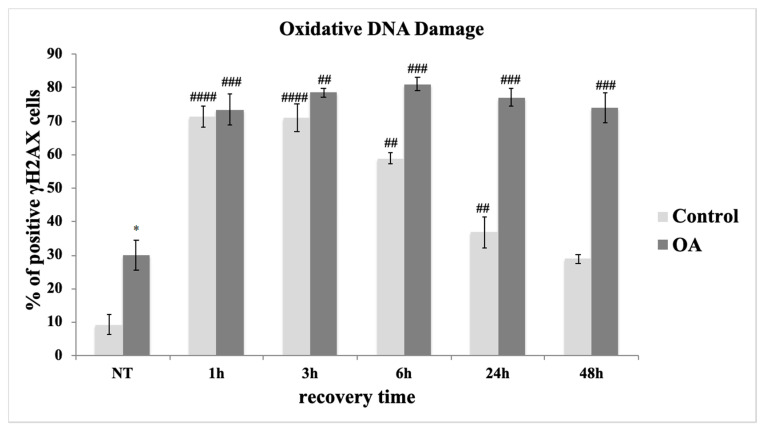
Osteoarthritic chondrocytes fail to repair oxidative DNA damage. Percentages of chondrocytes with DNA damage from three control and three OA patients samples evaluated by staining with the γH_2_AX antibody under normal conditions (No Treatment—NT) and at different time points of recovery period from the oxidative treatment (1 h, 3 h, 6 h, 24 h, and 48 h). Nuclei bearing >5 foci stained with γH2AΧ were counted as positive. Values shown are the means ± S.E. * *p* < 0.05 vs. control cells and ## *p* < 0.01, and ### *p* < 0.001, #### *p* < 0.0001 vs. NT. *p* values were calculated using the Student *t*-test.

As mentioned earlier, exposure to H_2_O_2_ results in the formation of SSB, which could turn into DSB in subsequent rounds of DNA replication. In order to evaluate DSB formation in cultured chondrocytes from three control and three OA patients after being exposed to exogenous oxidative insult, the presence of the phosphorylated form (on Ser139) of histone variant H2AX (γH2AX) was microscopically analysed. As shown in Figure 3, a remarkable increase in γH_2_AΧ-labelled lesions was observed at 1 h and 3 h post-treatment with H_2_O_2_, which started to decrease after 6 h of recovery time in the control cells. A decrease back to almost basal levels of damage was observed 48 h after OS-treatment in the control cells. In contrast, chondrocytes of OA individuals exhibited a high rate of damage 1 h after treatment with H_2_O_2_, which remains high even after 48 h of recovery time, implying the inability of OA cells to repair this damage (Figure 3). This result reaffirmed our previously reported data on the DNA damage repair failure of OA cells [7] while also extending the range of time-points assessed.

**Figure 4:**
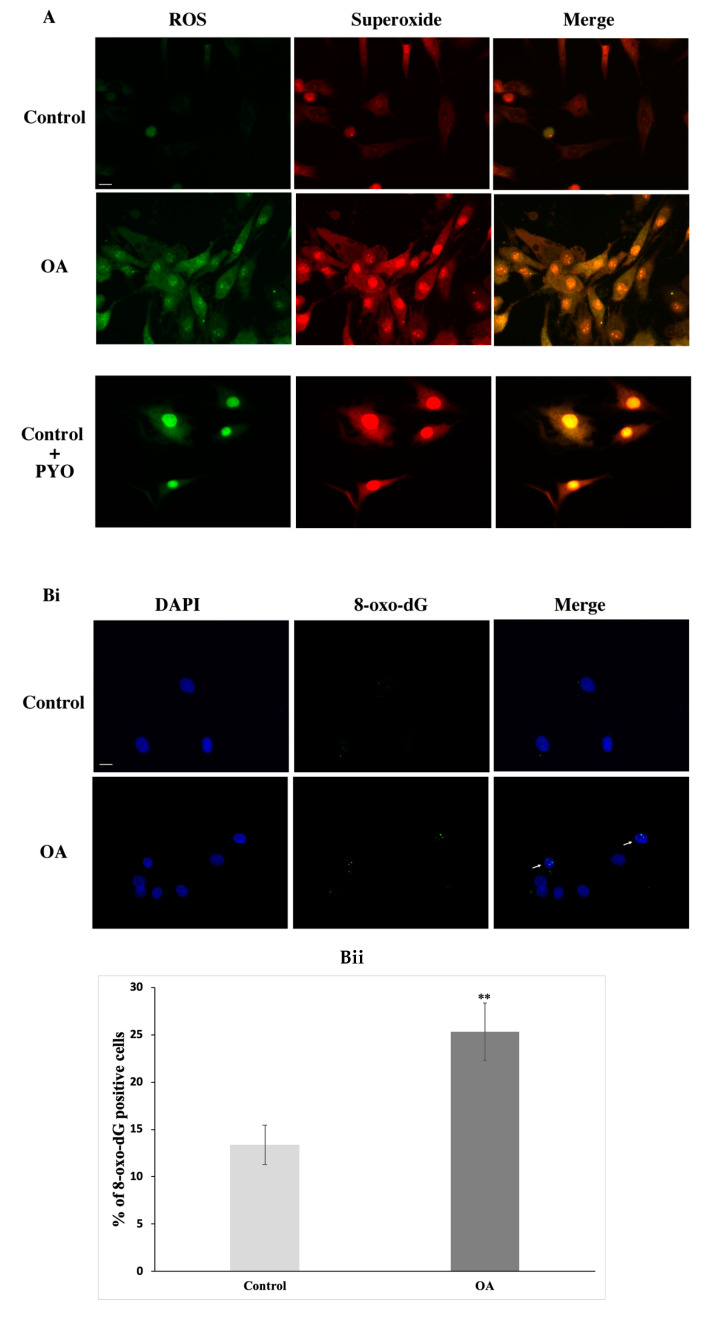

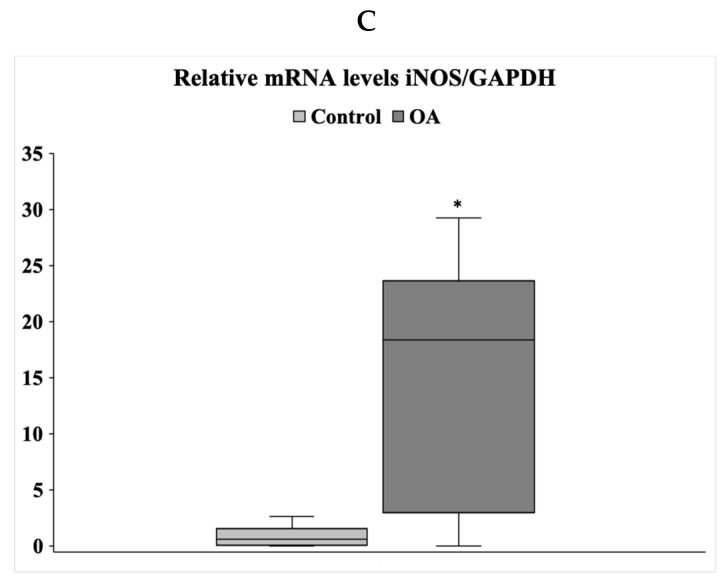
The osteoarthritic chondrocytes had elevated levels of oxidative stress even before the exposure to exogenous oxidative insult. (**A**) Characteristic images of control and OA chondrocytes (from three control and three OA patients samples, see method described in Materials and Methods) where total ROS and superoxide levels were analysed under normal conditions (Control-OA) and after pyocyanine was added to the control cells in order to serve as the positive control (Control + PYO). Images were captured with the 40× objective lens of the fluorescent microscope used. Scale bar 25 μm. (**Bi**) Oxidative DNA lesions were assessed by staining with 8-oxo-dG antibody and the appropriate secondary antibody (green). Nuclei bearing >5 foci stained with 8-oxo-dG were counted as positive. Nuclei were stained with DAPI (blue). Images were captured with the 40× objective lens of the fluorescent microscope used. Arrows point at nuclei having characteristic green foci indicative of the formation of 8-oxo-dG. Scale bar 25 μm. (**Bii**) Percentages of chondrocytes’ nuclei bearing 8-oxo-dG lesions from three control and three OA patients samples (Control-OA). Values shown are the means ± S.E. ** *p* < 0.01 vs. control cells. *p* values were calculated using the Student *t*-test. (**C**) Box blot demonstrating the quantification of relative mRNA levels of iNOS in the control and OA chondrocytes. The horizontal lines inside the boxes denote the medians and the horizontal borders of the boxes the interval between the 25th and 75th percentiles. The whiskers show the minimal and maximal values. Analysis was performed using chondrocytes from four healthy donors and four OA patients. * *p* < 0.05 vs. control. *p* values were calculated using the Mann–Whitney U test.

This result, besides showing the inability of OA chondrocytes to recover from the exogenous oxidative insult, also demonstrated a statistically significant higher number of γH2AX-positive OA cells compared to the control cells, even before the administration of H_2_O_2_ (Figure 3, NT). These DSBs could be the result of elevated oxidative pressure on chondrocytes in the OA joint as mentioned earlier [4]. In order to explore the latter, we analysed the oxidative stress (total ROS and superoxide levels) as well as the levels of 8-oxo-dG, a major marker of oxidative DNA damage as mentioned earlier, in OA and control chondrocytes under normal conditions. As shown, OA chondrocytes demonstrated dramatically higher levels of both total ROS (green) and superoxide (orange) when compared to control cells (Figure 4A) as well as a statistically significant higher percentage of cells bearing 8-oxo-dG foci (Figure 4Bi,Bii). It has been previously proven that elevated amounts of ROS could be the result of excessive NO production as it inhibits the mitochondrial OXPHOS chain. NO is synthesised by at least four isoforms of the NOS enzyme with inducible NOS (iNOS), being the only one responsible of generating micromolar levels of NO, therefore inducing a reactive nitrogen species (RNS) burst that could result in DNA damage [32]. Since, other studies have also found that caveolin-1 has a close link with iNOS activity and NO production, we, therefore, aimed at analysing iNOS mRNA levels in chondrocytes from healthy individuals as well as from OA patients. As Figure 4C shows, we found that OA chondrocytes have a statistically significant higher amount of iNOS mRNA levels as compared to the control cells.

#### 3.3.2. Assessment of Caveolin-1 Localization in Control and OA Chondrocytes after OS Treatment

**Figure 5:**
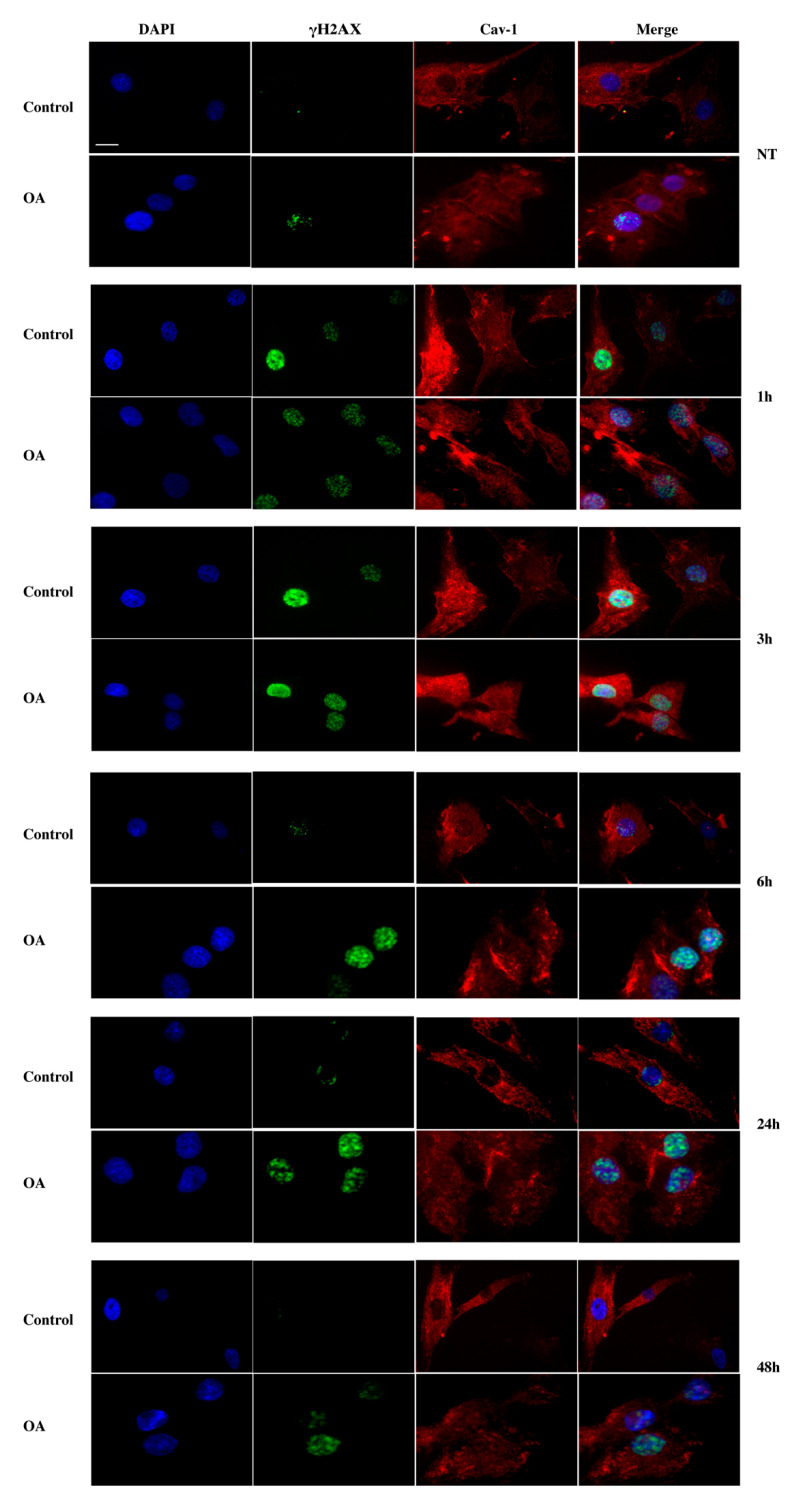
Translocations of caveolin-1 in and out of the nucleus in response to DNA damage appearance and restoration. Characteristic images of control and OA chondrocytes where caveolin-1 localization was evaluated by staining with anti-cav-1 antibody and the appropriate secondary antibody (red) under normal conditions (No Treatment—NT) and at 1, 3, 6, 24, and 48 h after being treated with H_2_O_2_. DNA damage was also assessed by staining with the γH2AX antibody and the appropriate secondary antibody (green). Nuclei were stained with DAPI (blue). Images were captured with the 40× objective lens of the fluorescent microscope used. Scale bar 25 μm.

**Figure 6:**
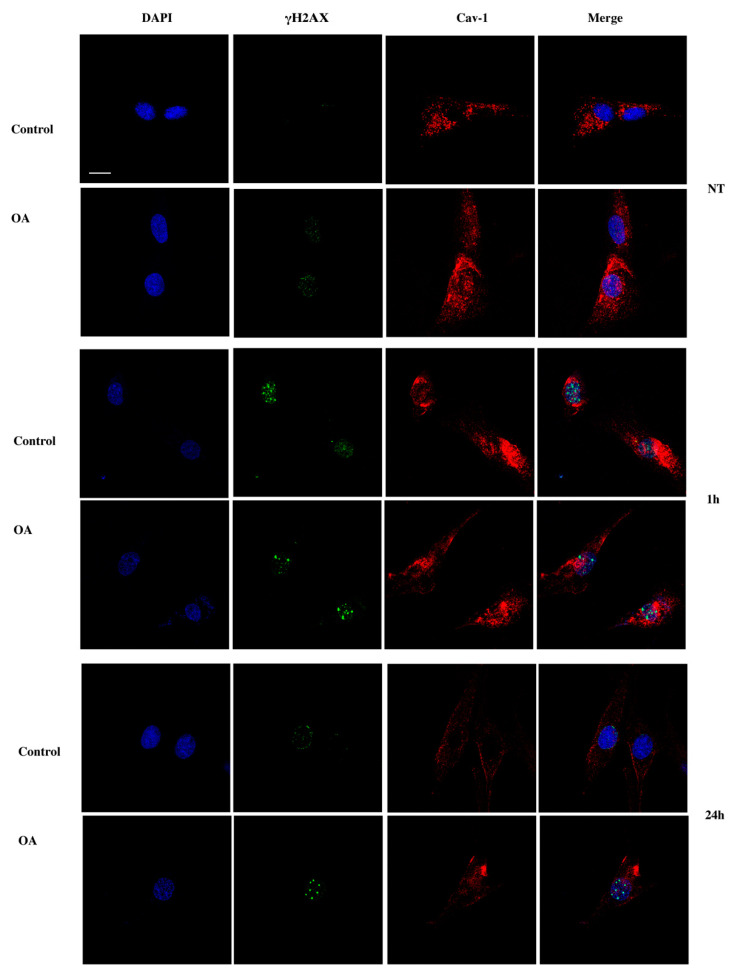
No translocation of caveolin-1 took place in osteoarthritic chondrocytes after exposure to exogenous oxidative insult. Characteristic images of the control and OA chondrocytes where caveolin-1 localization was evaluated by staining with anti-cav-1 antibody and the appropriate secondary antibody (red) under normal conditions (No Treatment—NT) and at 1 h and 24 h after being treated with H_2_O_2_. DNA damage was also assessed by staining with the γH2AX antibody and the appropriate secondary antibody (green). Nuclei were stained with DAPI (blue). Images were captured with the 60× objective lens of the ZEISS LSM 800 confocal microscope with Z stacks. Scale bar 25 μm.

Given the upregulation in the levels of caveolin-1 in response to DNA damage, aside from calculating DNA lesions, we also aimed at evaluating caveolin-1 subcellular localization in the control and OA chondrocytes with immunofluorescent microscopy. Interestingly, even before the exposure of the cells to OS, caveolin-1 showed a different localization pattern between normal and OA cells: in cells derived from healthy individuals, caveolin-1 was mainly found at the plasma membrane and cytoplasm, whereas in OA, chondrocytes were also found in the nucleus (Figure 5, NT). In addition, in the control cells at 1–6 h post-treatment, caveolin-1 was also found to be localized in the nucleus (Figure 5, 1 h, 3 h, 6 h) and after 24 h in recovery, it returned to its original localization (membrane-cytoplasm) (Figure 5, 24 h, 48 h). In contrast, in OA cells, no such translocations of caveolin-1 were observed at any time-point (Figure 5, NT, 1 h, 3 h, 6 h, 24 h, 48 h). In order to confirm translocation of caveolin-1 in and out of the nucleus in response to DNA damage appearance and restoration, the same experiment was performed and images were captured using confocal microscopy. Figure 6 verifies that, unlike control chondrocytes, in osteoarthritic chondrocytes, no translocation of caveolin-1 takes place after exposure to H_2_O_2_. Even though immunofluorescence analysis is not a quantitative method, a slightly higher intensity of caveolin-1 levels in OA cells at 1 h and 3 h (Figure 5) after treatment could be observed, which could be interpreted as opposing our above reported quantification of protein expression levels of caveolin-1 using western blotting (Figure 2C). We believe that, although the failure of OA cells to properly translocate caveolin-1 in and out of the nucleus is unquestionable, this apparent higher expression of caveolin-1 could represent the OA chondrocytes’ attempt to mobilize caveolin-1 in response to H_2_O_2_ induced DNA damage.

**Figure 7:**
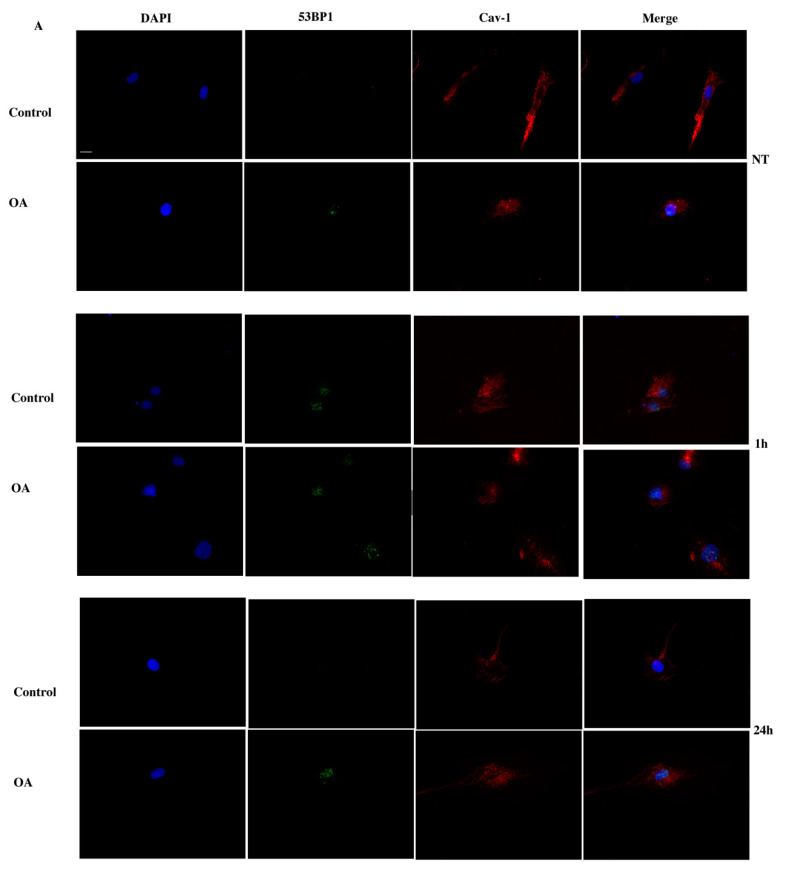

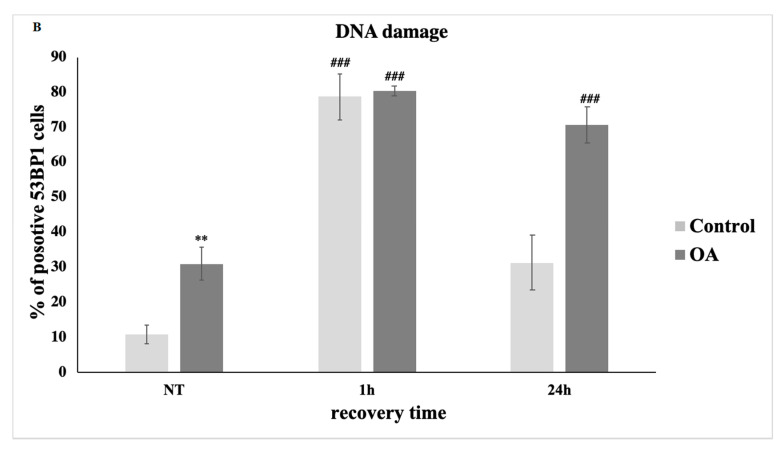
No translocation of caveolin-1 in osteoarthritic chondrocytes resulted in no restoration of damage. (**A**) Characteristic images of the control and OA chondrocytes where caveolin-1 localization was evaluated by staining with the anti-cav-1 antibody and the appropriate secondary antibody (red) under normal conditions (No Treatment—NT) and at 1 h and 24 h after being treated with H_2_O_2_. DNA damage was assessed by staining with the 53BP1 antibody and the appropriate secondary antibody (green). Nuclei were stained with DAPI (blue). Images were captured with the 40× objective lens of the fluorescence microscope. Scale bar 25 μm. (**B**) Percentages of chondrocytes with 53BP1 foci from three control and three OA patients samples evaluated by staining with the 53BP1 antibody under normal conditions (No Treatment—NT) and at different time points of the recovery period from the oxidative treatment (1 h and 24 h). Nuclei bearing >5 foci stained with 53BP1 were counted as positive. Values shown are the means ± S.E. ** *p* < 0.05 vs. control cells and ### *p* < 0.001 vs. NT. *p* values were calculated using the Student *t*-test.

DSB after the exposure to H_2_O_2_ were also evaluated using the 53BP1 specific antibody since, as mentioned earlier, these DNA lesions promote the phosphorylation of 53BP1, that, together with γH2AΧ, signals the initiation of the repair of this damage [20]. Similarly, to γH2AΧ analysis, 53BP1 foci assessment confirmed the inability of OA cells to repair the damage even 24 h after exposure to the oxidative insult (Figure 7A,B). Once again, it was evident that caveolin-1 translocation in and out of the nucleus coincided with the appearance and resolution of 53BP1 foci in the control cells, whereas no such translocation took place in OA cells (Figure 7A).

**Figure 8:**
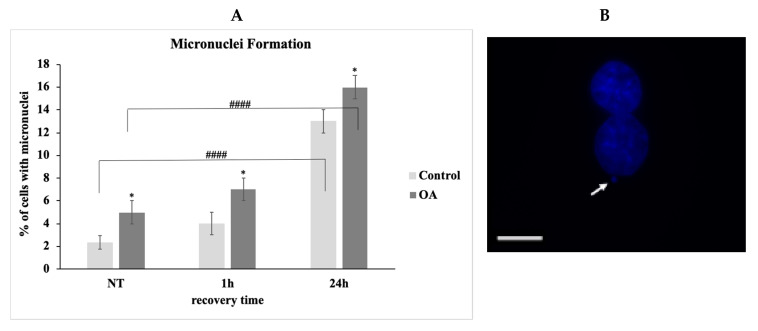
No restoration of OS-induced DNA damage resulted in the formation of a higher number of micronuclei in OA chondrocytes. (**A**) Percentages of chondrocytes from three control and three OA patients samples evaluated for the presence of micronuclei under normal conditions (No Treatment—NT) and at different time points of recovery period from the oxidative treatment (1 h and 24 h). Values shown are the means ± S.E. * *p* < 0.05 vs. control cells and #### *p* < 0.001 vs. NT. *p* values were calculated using the Student *t*-test. (**B**) Characteristic images of a micronucleus (arrow) in OA chondrocytes. Nuclei were stained with DAPI (blue). Images were captured with the 40× objective lens of the fluorescence microscope. Scale bar 25 μm.

We additionally examined the formation of micronuclei as a measure of genomic integrity and proper DNA repair, since micronuclei are known to form when cells enter mitosis with unresolved DNA damage [33]. The percentage of OA chondrocytes bearing micronuclei (Figure 8B) were statistically significantly higher than the control chondrocytes at all time points (Figure 8A), which further indicated their inability to restore OS-induced DNA damage.

**Figure 9:**
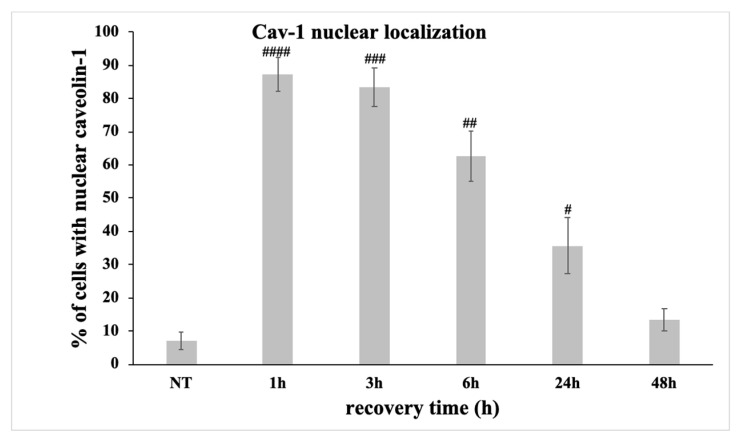
The percentage of control chondrocytes with nuclear caveolin-1 increased after oxidative insult and decreased back to basal levels for 48 h post-treatment. Percentages of control chondrocytes (three different control samples) with nuclear localization of caveolin-1 under normal conditions (No Treatment—NT) and at different time points of the recovery period from the oxidative treatment (1 h, 3 h, 6 h, 24 h, and 48 h). # *p* < 0.05, ## *p* < 0.01, and ### *p* < 0.001, #### *p* < 0.0001 vs. NT. *p* values were calculated using the Student *t*-test.

The previous results (Figure 3, Figure 5, Figure 6, Figure 7, and Figure 8) implied a causal relationship between the inability of caveolin-1 to properly translocate to the nucleus after oxidative stress-induced DNA damage and the failure of OA chondrocytes to restore this damage. We therefore analysed the percentage of cells from healthy individuals (control) in which caveolin-1 was found in the nucleus—aside from its membrane/cytoplasm localization—at all time-points (under normal conditions-NT and at 1, 3, 6, 24, and 48 h after the exposure of H_2_O_2_) using immunofluorescent microscopy. As expected, there was a correlation between the number of γH2AΧ-positive and 53BP1-positive control cells (Figure 3 and Figure 7B, respectively) and the control cells with nuclear localization of caveolin-1 (Figure 9) at all time points, which further suggests a role for caveolin-1 in DNA damage signalling and/or repair. This correlation also indicates that the observed here failure of OA chondrocytes to spatiotemporally regulate caveolin-1 could be accounted for their inability to repair the OS-induced DNA damage.

**Figure 10:**
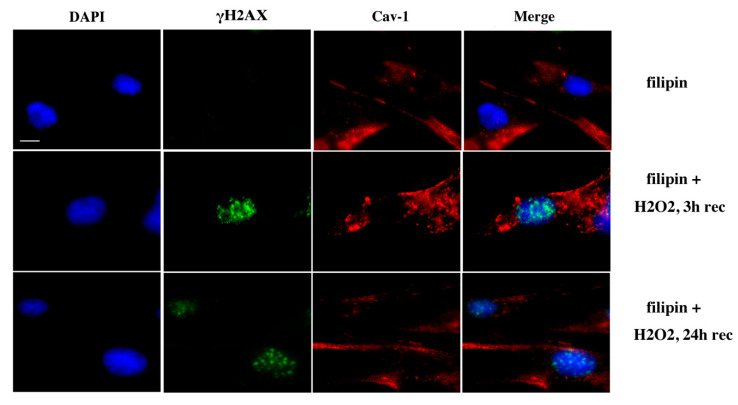
Filipin prevents caveolin-1 nuclear translocation. Characteristic images of caveolin-1 localization evaluated by anti-cav-1 antibody (red) in control chondrocytes treated with filipin for 24 h (filipin) before exposing cells to H_2_O_2_ and allowing them to recover for 3 h (filipin + H_2_O_2_, 3 h rec) and 24 h (filipin + H_2_O_2_, 24 h rec). DNA damage (γH2AX, green) was also assessed and nuclei were stained with DAPI (blue). Images were captured with the 100× oil immersion objectives of the fluorescent microscope used. Scale bar 25 μm.

**Figure 11:**
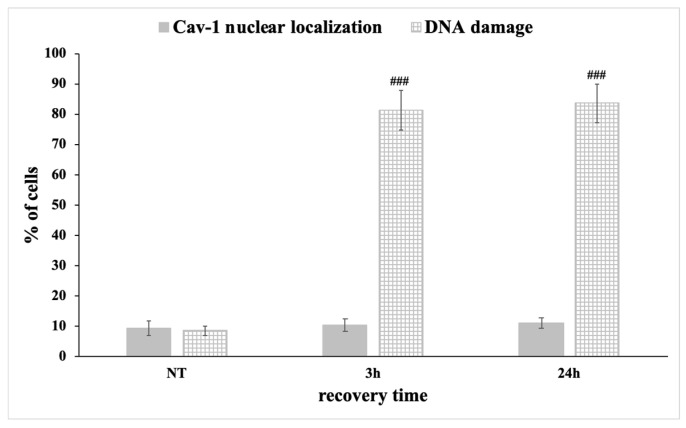
Prevention of caveolin-1 nuclear translocation inhibits DNA lesions restoration. Percentages of three different control chondrocytes pre-treated with filipin for 24 h with nuclear localization of caveolin-1 and DNA damage lesions (>5 foci stained with γH2AΧ) under normal conditions (No Treatment—NT) and at different time points of recovery from the oxidative treatment (3 h, 24 h). Values shown are the means ± S.E. ### *p* < 0.001 vs. NT. *p* values were calculated using the Student *t*-test.

In order to further strengthen this suggestion, we used filipin, a chemical inhibitor of caveolin-1, which is known to bind cholesterol in the plasma membrane and impair caveolae internalization and invagination and therefore impedes caveolin-1 translocation [34]. Specifically, chondrocytes from three healthy individuals (control) were pre-treated with filipin for 24 h before the exposure of the cells to OS (H_2_O_2_ 200 μΜ, 30 min). Cells were then left to recover for 3 to 24 h in medium containing filipin. As shown in Figure 10 and Figure 11, a low percentage of the cells pre- treated with filipin for 24 h demonstrated caveolin-1 in the nucleus (9.33 ± 2.3) and DNA damage was at basal levels (8.66 ± 1.52) (Figure 10, upper panel, and Figure 11). As expected, there was no significant increase in the percentage of cells with caveolin-1 in the nucleus at 3 h (10.33 ± 2.08) or at 24 h (11 ± 1.73) after the exposure to H_2_O_2_ (Figure 10, middle/lower panel, and Figure 11). Interestingly, DNA damage 3 h after the exposure to OS reached higher levels (81.33 ± 6.65) compared to the levels of damage calculated when exogenous OS was done without filipin pre-treatment (71.33 ± 3.21/Figure 3). More importantly, as shown in the same figures, the control cells treated with filipin failed to restore DNA damage even 24 h after treatment with H_2_O_2_ (83.66 ± 6.5). The latter suggests that the prevention via filipin of caveolin-1 translocation to the nucleus could be responsible for the failure of chondrocytes to repair OS-induced DNA damage.

## 4. Discussion

The inability of OA chondrocytes to repair DSB that result from the exposure of cells to oxidative stress has been recently reported by our group [7]. Here, we further confirm this observation and provide novel evidence for the role of caveolin-1 in the OA chondrocytes’ impaired response to genotoxic stress. Specifically, we showed that caveolin-1 transcriptional and protein levels under normal conditions are upregulated in OA chondrocytes as compared to cells derived from healthy individuals. Caveolin-1 overexpression is correlated with upregulated levels of senescence marker p16 in OA chondrocytes. This caveolin-1 overexpression could be accounted for the, observed here, failure of OA cells to further upregulate its levels in response to OS and other types (bleomycin) of genotoxic stress; in contrast, in the control cells, caveolin-1 levels were upregulated immediately after the exposure to stress(es) and returned to its basal levels after 6–24 h in recovery. Furthermore, the transient modifications in caveolin-1 levels coincided with the appearance and repair of DNA damage lesions in the control cells, providing evidence for caveolin-1 involvement in DNA damage signalling/repair mechanism(s). The latter observation urged us to also explore the localisation pattern of caveolin-1 in healthy and OA chondrocytes under normal conditions as well as under OS. We detected that under normal conditions, caveolin-1 localized at the membrane/cytoplasm of control cells while in OA cells, it was also undoubtedly found in the nucleus as well as at the other cellular compartments. Interestingly, in the control cells, caveolin-1 translocated to the nucleus after exposure to OS, and after 24 h in recovery it was again found at its original locations (membrane/cytoplasm). The calculation of the percentages of γH2AΧpositive as well as 53BP1-positive control cells and the percentage of cells with nuclear localization of caveolin-1 at all time points revealed a strong correlation between caveolin-1 translocation in and out of the nucleus and DSB appearance and restoration. More importantly, pre-treatment of control cells with filipin prevented caveolin-1 nuclear translocation and compromised the ability of these cells to resolve the OS-induced DNA damage. In contrast, in OA chondrocytes, no nuclear translocation of caveolin-1 was observed following exogenous oxidative insult, which could account for the impaired response of OA cells to genotoxic insults. The summary of the above observations and proposed mechanistic explanation(s) is depicted in the Graphical Abstract.

Several studies have demonstrated limited antioxidant ability of OA cartilage, suggesting that redox imbalance contributes in OA pathogenesis [35]. OS results in damage to the cell membrane and macromolecules, mainly proteins and nucleic acids [36]. In fact, as we previously demonstrated [7] and reaffirmed here, OA chondrocytes failed to repair OS-produced DSB to the DNA. The persistence of such damage could lead to OA chondrocyte senescence, especially since telomere shortening in OA, reported in previous studies [37], has been questioned by other studies [38]. As chondrocyte senescence is also implicated in disease onset and progression [5], the failure of OA chondrocytes to repair oxidative damage is becoming a central feature of the OA aetiopathogenic atlas. In fact, several senescent features have been recognized in OA chondrocytes such as senescence-associated β-galactosidase activity [39] and increased p16ink4a expression [40]. Additionally, it has been recently shown that transplantation of senescent cells into a healthy knee joint in mice gives rise to OA-related features [41]. Even more, Jeon and colleagues demonstrated that selective elimination of senescent cells from the joint delays the onset of OA [42]. As normal articular chondrocytes rarely replicate, it is safe to consider their senescence as the outcome of stress rather than replication exhaustion [38]. In fact, high shear stress on articular surfaces can lead to ROS formation and therefore OS, which promotes senescence [43].

Caveolin-1 has emerged as an important regulator of oxidative stress-induced senescence of several cell types including fibroblasts [8] and articular chondrocytes [16]. The aforementioned reports demonstrated an increase in the levels of caveolin-1 after oxidative stimulation, whereas the opposite effect was demonstrated in cardiomyocytes [44] and myoblasts [45]. These controversial results on caveolin-1 expression levels post-OS treatment could simply reflect the cell type specificity of the phenomenon. Furthermore, Dai and his colleagues, who performed H_2_O_2_ experiments in OA chondrocytes similar to ours, reported a sustained increase in caveolin-1 mRNA and protein levels along with an onset of senescence features. However, unlike our approach, Dai and colleagues exposed the cells to prolonged (24 h) OS and did not include any comparisons with chondrocytes derived from healthy individuals. The acute oxidative insult performed here might be closer to the fluctuations of oxygen tension due to OA related ischemia–reperfusion phenomenon and constant abnormal mechanical strains present in the OA joint [46]. Additionally, the comparison we performed with the control cells revealed the already elevated levels of caveolin-1 in OA chondrocytes under normal conditions, which can explain the OA cells’ inability to properly regulate caveolin-1 levels in response to acute OS. Moreover, it has been previously demonstrated that upon OS, caveolin-1 sequesters the p53 specific ligase, Mdm2, and this prevents p53 proteasomal degradation, leading to its upregulation and induction of premature senescence [47]. Our results add novel parallel elements to this previously reported role of caveolin-1 as a key player in the pleiotropic nature of cellular senescence [48].

It is worth mentioning that it has been previously reported that the activity of iNOS, the production of NO as well as the levels of intracellular ROS are elevated in OA as well as in normal chondrocytes treated with H_2_O_2_ [49]. We confirmed the elevated ROS levels (as well as augmented oxidative DNA lesions) and iNOS mRNA levels in OA cells under normal conditions as compared to control cells. What is interesting is the relationship between caveolin-1 levels, iNOS activity, and ROS formation; it has been reported that Cav-1 loss has a close link with the increased NO synthase (NOS) activity and NO production [32]. On the other hand, overexpression of Cav-1 generated significantly higher superoxide anion level in lung cancer H460 cells while caveolin-1 specific shRNA transfection resulted in decreased superoxide generation [50]. The latter as well as our observations here point at a heterogeneous effect of caveolin-1 overexpression/silencing onto oxidative stress modulation, while nevertheless revealing a rather interesting bidirectional regulatory relationship between caveolin-1 and oxidative stress.

The OA-related impaired regulation of caveolin-1 in response to oxidative stress that we report here was also evident when localization experiments were performed. Caveolin-1 is located predominantly at the membrane, but also in the Golgi, the endoplasmic reticulum, and at cytosolic locations [51]. In accordance with these previous reports, in our experiments, caveolin-1 was found to be localised at these sites in the control cells under normal conditions, and a clear translocation to the nucleus was observed following OS stimulation. Similarly to our observations, a previous study has reported nuclear localisation of caveolin-1 in human diploid fibroblasts following oxidative exposure [52]. Even more, the strong correlation we observed here in the control cells between caveolin-1 translocation in and out from the nucleus and DSB appearance and restoration implies an implication of caveolin-1 in DNA damage repair/signalling. In fact, it was previously shown that caveolin-1 plays a critical role in DNA damage repair as it is involved in regulating important molecules of homologous recombination (HR) and non-homologous end joining (NHEJ) mechanisms [19]. Specifically, Zhu et al. demonstrated that cavolin-1 regulates ATM activation as well as BRCA1 accumulation on γH2AΧ foci in ionizing-radiation (IR)-treated cells, which is a crucial step in HR. Moreover, in the same study, it was shown that knockdown of caveolin-1 inhibited the IR-activated phosphorylation of DNA-PK, a key molecule in the NHEJ system [19]. In support, it was demonstrated that under oxidative stress, caveolin-1 gets phosphorylated and this is essential for the internalisation and transportation of EGFR (Epidermal Growth Factor Receptor) to the nucleus, where it forms a complex with DNA-PK [53,54]. The role of caveolin-1 as a possible sensor and mediator of the DNA damage repair process was further supported by the experiments of Hossain and colleagues, who showed that upon genotoxic stress, the tyrosine kinase TIE2 phosphorylates caveolin-1 in the caveolae, which facilitates the co-translocation of both proteins to the nucleus. The same study demonstrated that filipin treatment prevents caveolin-1 translocation to the nucleus and this was associated with significant radio-sensitisation of malignant glioma cells [55]. Here, aside from supporting the extremely limited reports describing the localization of caveolin-1 in the nucleus [55,56], we also demonstrated that the same treatment with filipin resulted in impairment of the control chondrocytes’ ability to restore OS-induced DNA damage. We therefore concluded that the nuclear localisation of caveolin-1 in OA cells even before the treatment with H_2_O_2_ could reflect (a) the fact that the osteoarthritic joint is an environment of elevated oxidative pressure [4,46] and (b) that the implication of caveolin-1 in DNA damage signalling and/or repair depends on its specific spatiotemporal regulation—possibly in a complex with DNA damage repair factor(s)—that is absent in OA cells.

Taken together, our data expand our knowledge regarding the implication of caveolin-1 in response to oxidative stress. Moreover, as numerous studies have implied that the age-related increase in ROS production in articular cartilage promotes the development of OA [57], the proven here, inability of OA chondrocytes to properly regulate the function(s) of caveolin-1 provides an underlying mechanistic feature to the OS-driven onset of this disease. The latter could prove beneficial for OA, and possibly other age-related diseases, management.

## 5. Conclusions

A series of studies have shown that oxidative stress (OS) occurs with ageing in the articular cartilage and this promotes the development of osteoarthritis (OA). We have previously demonstrated that this OS also leads to mitochondrial dysfunction and autophagy impairment, which will, in turn, amplify OS-mediated macromolecular damage, comprising a vicious loop that promotes senescence [6,7]. Here, we demonstrate that the onset of senescence in OA chondrocytes is accompanied, not only by OS-induced DNA damage, but also by an increase in the levels of caveolin-1, which is also found to be localised in the nucleus, aside from its normal localisation at the membrane/cytoplasm. Moreover, no upregulation of caveolin-1 levels and no translocation in/out of the nucleus of caveolin-1 was observed after exposure of OA cells to exogenous acute oxidative treatment (H_2_O_2_), as seen in chondrocytes from healthy individuals. We therefore propose that the observed here OA cells’ inability to restore OS-induced double strand breaks (DSB) onto the DNA is caused by defects in translocation (and possibly formation) of caveolin-1/DNA damage repair factors complex to the nucleus. This suggests a caveolin-1 associated impairment of OA cells’ redox defence that could contribute to the aetiopathogenesis of the disease.
